# Temperature-Triggered Enzyme Immobilization and Release Based on Cross-Linked Gelatin Nanoparticles

**DOI:** 10.1371/journal.pone.0047154

**Published:** 2012-10-10

**Authors:** Zhenhai Gan, Ting Zhang, Yongchun Liu, Daocheng Wu

**Affiliations:** 1 Key Laboratory of Biomedical Information Engineering of Education Ministry, School of Life Science and Technology, Xi’an Jiaotong University, Xi’an, P. R. China; 2 Scientific Research Center, The Second Affiliated Hospital, School of Medicine, Xi’an Jiaotong University, Xi’an, P. R. China; King Abdullah University of Science and Technology, Saudi Arabia

## Abstract

A glucoamylase-immobilized system based on cross-linked gelatin nanoparticles (CLGNs) was prepared by coacervation method. This system exhibited characteristics of temperature-triggered phase transition, which could be used for enzyme immobilization and release. Their morphology and size distribution were examined by transmission electron microscopy and dynamic light scattering particle size analyzer. Their temperature-triggered glucoamylase immobilization and release features were also further investigated under different temperatures. Results showed that the CLGNs were regularly spherical with diameters of 155±5 nm. The loading efficiencies of glucoamylase immobilized by entrapment and adsorption methods were 59.9% and 24.7%, respectively. The immobilized enzyme was released when the system temperature was above 40°C and performed high activity similar to free enzyme due to the optimum temperature range for glucoamylase. On the other hand, there was no enzyme release that could be found when the system temperature was below 40°C. The efficiency of temperature-triggered release was as high as 99.3% for adsorption method, while the release of enzyme from the entrapment method was not detected. These results indicate that CLGNs are promising matrix for temperature-triggered glucoamylase immobilization and release by adsorption immobilization method.

## Introduction

Enzyme immobilization is a technique in which an enzyme is made to attach to an inert material. It can provide increased resistance for the immobilized enzyme to the changes in conditions such as pH value or temperature [1]–[5]. Compared with free enzyme systems, immobilized enzymes offer the advantages of having batch or continuous processes operations, rapid termination of reactions, controlled product formation, ease of removal from the reaction mixture, and adaptability to various engineering designs [6]. Thus, enzyme immobilization has been widely used in analysis [7], medicine [8], food [9], chemical industry [10], [11] and biotechnology [12], [13] areas. For example, in recently reported studies, Terres et al. immobilized chloroperoxidase on silica matrix both physically and covalently for the biocatalytic desulfurization of fossil fuels [14]. Laveille et al. reported mesoporous silica immobilization for hemoglobin. In this system, the adsorbed hemoglobin was carried out for the detection of polycyclic aromatic hydrocarbons pollutant in the presence of H_2_O_2_. The catalytic performance of hemoglobin was enhanced for the real sample treatment under the pH range 6.5–8.5, while optimum pH for free hemoglobin is 5.0 [15].

Enzyme immobilization may be broadly divided into two main categories. One is based on the formation of covalent bonds between enzyme and matrix. The other method is physical method which is based on molecular interactions between the enzyme and support, such as adsorption and entrapment. Covalently immobilized enzyme showed an inactivating catalytic ability because of the reaction between groups necessary for enzyme activity or groups responsible for tertiary structure [16], [17]. Comparatively, enzyme immobilization by adsorption appears to offer better commercial potential because it is simpler, less expensive, and retains higher catalytic activity [18], [19]. With this method, no coupling reagent or reactive group of any amino acid residue of the enzyme is required to form specific covalent bonds with the supports. However, several studies have found that physically adsorbed enzymes on most supports were generally not strong enough, causing slow enzyme leakage during washing, operation and reaction processes [20]–[22]. Hence, strong adsorption between the enzyme and support should be achieved to prevent enzyme desorption from immobilization supports [23]. Furthermore, enzymes immobilized on solid-supports refer to two-phase systems with inherent mass transfer limitations. Immobilized enzyme systems suffer from producing unfavorable effects on their overall catalytic performances, such as the catalysis happened beyond the optimum temperature of enzyme or enzyme could not be released at desirable time [1], [24], [25]. Thus, developing a new strategy for enzyme immobilization is urgently needed to address the above-mentioned problems.

Smart hydrogels, which have the capability respond to small external stimulus changes, have attracted substantial attention in recent years, particularly in the pharmaceutical and material fields [26]. Many physical and chemical stimuli have been applied to induce various responses of the smart hydrogel systems. The physical stimuli include temperature, electric fields, solvent composition, light, pressure, sound and magnetic fields, while the chemical or biochemical stimuli include pH, ions and specific molecular recognition events [27]–[29]. If these systems were used for enzyme immobilization, enzyme would be released at needful time and optimum conditions under which enzyme could perform its highest catalysis ability. Among the smart hydrogels available, temperature sensitive hydrogels are probably the most commonly studied, especially in drug delivery and enzyme immobilization research. Qiu et al. summarized a series of temperature-sensitive systems, including poly(*N*-isopropylacrylamide), poly(*N*, *N*-diethylacrylamide), poly(ethylene oxide) and poly(propylene oxide) [26]. However, the biocompatibility of currently available polymers for these temperature sensitive hydrogels is not satisfactory, which limits their applications in fields such as the food and beverage industry. The natural biopolymer gelatin, obtained through partial hydrolysis of collagen, exhibits good biocompatibility, non-toxic biodegradation *in vivo* and readily excreted products. It also features highly effective drug encapsulation and can be fabricated into various forms of carriers for controlled drug and DNA delivery [30], [31]. Based on our previous research, a cross-linked gelatin nanoparticles (CLGNs) system prepared by coacervation method showed the ability to shrink at room temperature and swell when the temperature increased. Thus, a temperature-sensitive system made of gelatin could provide a promising strategy for enzyme immobilization and release, which has the characteristic of controlled-release at desirable time and optimum temperature for the enzyme. To the best of our knowledge, few reports on the gelatin hydrogel system for the enzyme immobilization existed and no controlled-release enzyme immobilization system based on gelatin hydrogel could be found [16], [32]–[34]. Glucoamylase is an industrially important enzyme used for the large-scale saccharification of malto-oligosaccharides into glucose and various syrups required in the food, beverages and fuel ethanol industry [23]. The suitable temperature for this enzyme is 40–70°C. Thus, its wide application in the biotechnology field is limited. Immobilize glucoamylase into a temperature-sensitive smart hydrogel system can either protect it from environmental damage when the temperature is beyond the suitable range, or release it when the temperature is suitable for the enzyme perform high catalytic ability, thereby yielding optimum application conditions.

**Figure 1:**
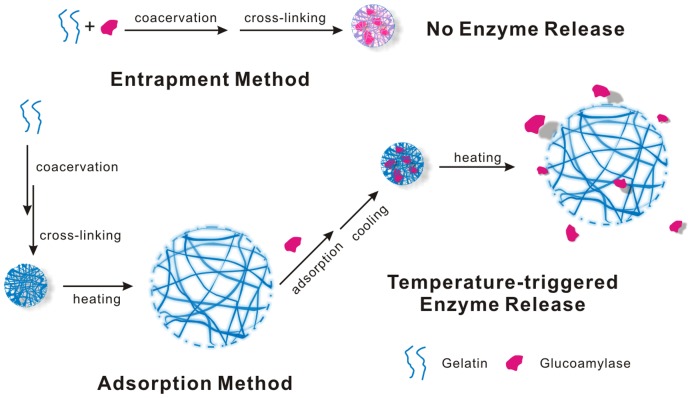
Schematic illustrations for the preparation of CLGNs, glucoamylase immobilization, and temperature-triggered enzyme release. The gelatin and glucoamylase were mixed together before the cross-linking step in the entrapment method. In the adsorption method, the enzyme was adsorbed after the formation of the CLGNs.

In this work, a strategy for designing a temperature-sensitive cross-linked gelatin nanoparticles-based immobilized enzyme system is proposed. Specifically, this system has the characteristics of temperature-triggered enzyme release ranging from 40 to 80°C, which is established on the swelling of the CLGNs as shown in Figure 1. Glucoamylase was selected as the model enzyme because of its appropriate activity temperature range around 40–70°C and the considerable academic interest it has gained [16], [17], [35], [36]. During glucoamylase immobilization, non-covalent physical adsorption and entrapment methods were compared based on their immobilization and release efficiencies. The results indicate that this kind of temperature-sensitive enzyme immobilization system may be a new technology for enzyme immobilization, and could provide a new and effective alternative for enzyme immobilization in biotechnology.

## Materials and Experiments

### Materials

Gelatin (Type B, 238–282 Bloom, isoelectric point 4.0–5.7) was purchased from Amresco Inc. (Solon, OH, U.S.A.). Tween-20 was obtained from Sigma-Aldrich Corporation (St. Louis, MO. U.S.A.). Glutaraldehyde was obtained from Sinopharm Chemical Reagent (Shanghai, China). Sodium sulfate, isopropanol, sodium pyrosulfite, sodium hydroxide, 3,5-dinitrosalicylic acid (DNS), and starch were analytical grade and used as purchased. Glucoamylase (EC 3.2.1.3) was purchased from Shanghai Kayon Biological Technology Co., Ltd. (Shanghai, China) and dissolved in acetate buffer (0.1 mol/L, pH 4.6). The concentration of the glucoamylase solution was determined to be 1.0 mg/mL using UV-Vis absorption spectra. Sodium salts with different anions used for the surface modification of the CLGNs were all analytical grade and used as purchased. The concentrations of these series solutions were dependent on the charge number of the anions to obtain the same ionic strengths for all these solutions. A 14KD dialyzer was purchased from Sino-American Biotechnology (Shanghai, China). All chemicals were used without further purification. Double-distilled water (18 MΩ cm) was used as solvent.

### Preparation of Cross-linked Gelatin Nanoparticles

The preparation method involved the process of coacervation with sodium sulfate and cross-linking by glutaraldehyde. In our previously published paper, the fluoric anion was used for the modification of the CLGNs in an ultrasound-triggered drug release system because fluoride anions have the ability to attract electrons and can establish hydrogen bonds between oxygen and nitrogen atoms in the gelatin nanoparticles [37]. In present study, a series of anions with different charge numbers, such as F^−^, Cl^−^, NO_3_^−^, CH_3_COO^−^, SO_4_^2−^ and PO_4_^3−^, were used to investigate the optimum temperature response property. Briefly, CLGNs were obtained as follows: 2.0 mL of 5% gelatin stock solution and 1.0 mL of anion solution with the same ionic strengths for surface modification was mixed together in a 50 mL beaker, followed by the addition of double-distilled water to the final volume of 10 mL. The mixture solution was stirred under 37°C for several minutes with the addition of 100 µL of Tween-20. A 20% (w/w) of sodium sulfate solution was added dropwise to the mixture until a small amount of precipitate formed. Isopropanol was then added dropwise to dissolve the precipitate by sodium sulfate and obtain a clear solution again. The temperature of the mixture was lowered to 25°C, and 0.1 mL glutaradehyde was introduced into the mixture as a cross-linking reagent. After 5 min stirring, 2.0 mL of 12% (w/w) sodium pyrosulfite was added to stop the cross-linking, and the system was maintained under continuous stirring for another 90 min. The final clear product was dialyzed with double-distilled water for 24 hours to remove the unreacted reagents and stored at 4°C prior to use.

### Characterization of Cross-linked Gelatin Nanoparticles

The temperature responses of the different anion modified CLGNs solutions were examined by immersing the solutions in a thermostated water bath with the temperature set to rise gradually. The temperatures and response times were recorded when the clear solutions turned translucent.

The morphology of the CLGNs was characterized by transmission electron microscopy (JEM-2000EX, JEOL, Tokyo, Japan) using phosphotungstic acid as negative staining reagent. TEM imag of glucoamylase immobilized CLGNs prepared by adsorption method was also taken (H-7650, Hitachi, Tokyo, Japan). Their sizes and size distributions were determined using dynamic light scattering (DLS, Malvern Zetasizer Nano ZS90, Malvern instruments Ltd., U.K.). The zeta potential of the CLGNs under different pH and temperature were also measured with the same instrument.

The temperature-triggered swelling behavior of the CLGNs was investigated at different temperatures, increasing from 25 to 60°C, then decreasing back to 25°C. The swelling ratio was calculated using following equation:
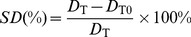
(1)


Here *SD*, *D*_T_, and *D*_T0_ are the swelling ratio, the diameter of CLGNs at certain temperature, and the diameter at 25°C, respectively.

The functional groups on the surface of the CLGNs were evaluated by a Fourier transform infrared spectrometer (FTIR, IR presitge-21, Shimazu, Kyoto, Japan). In a typical procedure, 0.25 mg of lyophilized CLGNs powder was mixed with IR-grade KBr (0.1 g) and pressed into a tablet form, and then the spectrum was recorded.

### Immobilization of Glucoamylase

In the present study, two strategies for glucoamylase immobilization were chosen. The first one was the entrapment of glucoamylase for immobilization. In this method, a certain volume of glucoamylase stock solution was added to the gelatin mixture before the addition of sodium sulfate at the coacervation step. The following steps were the same as the preparation of CLGNs mentioned above. After dialysis for 24 hours, the mixture was stored at 4°C prior to use. The other method was adsorption of the glucoamylase. Briefly, after the preparation of pure CLGNs as described above, the dialyzed nanoparticles solution was lyophilized to powder form. A certain amount of gelatin powder was added to 3 mL of glucomylase solution and stirred at different temperature (25, 37, and 60°C) for four hours, and then at 25°C for one hour. The glucoamylase adsorbed CLGNs solution was also stored at 4°C prior the measurement of temperature-triggered release of enzyme.

### Efficiency of Glucoamylase Immobilization

The activities of glucoamylase were assayed by the measurement of glucose produced by the hydrolysis of starch solution catalyzed by glucoamylase. The amount of glucose was determined using the DNS method according to reported reference [23]. Briefly, 0.5 mL of diluted free enzyme or the solution containing immobilized glucoamylase were added to 0.5 mL of soluble starch solution which contained 1.0% of (w/v) soluble starch in water as the substrate, followed by the addition of 0.5 mL of acetate buffer solution (0.1 mol/L, pH 4.6) under different temperatures. After stirring for 15 min, the reaction was stopped by the addition of 0.5 mL of NaOH solution (10% w/v). The glucose content in the reaction medium was then determined using the DNS method by the measurement of the UV-Vis absorption spectra at the wavelength of 520 nm. Each sample was measured three times, after that the average value was used for further calculations.

To determine the amount of immobilized glucoamylase, the mixture solution for the glucoamylase immobilization, both entrapped and adsorbed, was centrifuged for 10 min at 18000 rpm for the sedimentation of CLGNs. The activities of glucoamylase in the suspensions were measured by DNS method. The efficiency of immobilization was calculated by the decreased glucoamylase activity in the suspension after the immobilization compared to the free enzyme solution used for immobilization according to following equation:
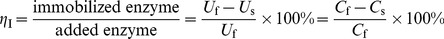
(2)


Here *η*_I_ is the efficiency of the immobilization. *U*_f_ and *U*_s_ are the glucoamylase activities of the free enzyme solution used for immobilization and the suspension after centrifugation, respectively. *C*_f_ and *C*_s_ are the corresponding glucose concentrations determined by the DNS method for the starch solution added the free enzyme solution and the suspension after centrifugation, respectively.

### Temperature-Triggered Glucoamylase Release

To determine the temperature-triggered glucoamylase release efficiency, the release of enzyme was carried out at different temperature. After centrifugation, the glucoamylase immobilized CLGNs sedimentation was washed three times with 0.5 mL acetate buffer solution (0.1 mol/L, pH 4.6) and centrifugation again. After the last washing, certain volumes of solution containing the enzyme immobilized CLGNs were added to series cuvettes, in which 0.5 mL of 1.0%(w/v) starch solution and acetate buffer (0.1 mol/L, pH 4.6) were previously mixed. These cuvettes were incubated under different temperatures for exactly 5 min for the release of glucoamylase. 0.5 mL of NaOH solution (10% w/v) was then added to stop the glucoamylase catalyzed hydrolysis of starch. The DNS method was used to determine the activity of the released glucoamylase as mentioned above. The release efficiency was calculated as the proportion of the released enzyme to the immobilized enzyme, which could be calculated according to the equation:
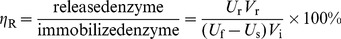
(3)


Here *η*_R_ is the release efficiency. *U*_r_ is the activity of the released glucoamylase. *V*_i_ and *V*_r_ are the volumes of the solutions in the enzyme immobilization and release steps, respectively. We set the *V*_i_ and *V*_r_ the same value in the experiment, then equation (3) could be simplified as following:
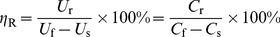
(4)


Here *C*_r_ referred to the glucose concentration in the release experiment and determined by DNS method.

To investigate the effect of temperature on the activity of glucoamylase at the immobilization and release steps, a certain volume of enzyme immobilized CLGNs solution was immersed in a thermostated water bath at 60°C for four hours to complete the release of glucoamylase. The solution was then centrifuged, and the activity of enzyme in the suspension was measured at different temperature using the DNS method as mentioned above. The free enzyme activities at different temperature were also measured for comparison.

### Statistical Analysis

One-way ANOVA was performed to determine the differences among the size distribution of CLGNs in the temperature-sensitive swelling experiment. The differences of glucoamylase immobilization and release efficiency at different temperatures were also determined. Newman–Keuls test was performed as post-hoc analysis for one-way ANOVA. Differences between the compared data with P-values <0.05 were considered statistically significant.

## Results and Discussion

### Temperature Response of Cross-linked Gelatin Nanoparticles

**Figure 2:**
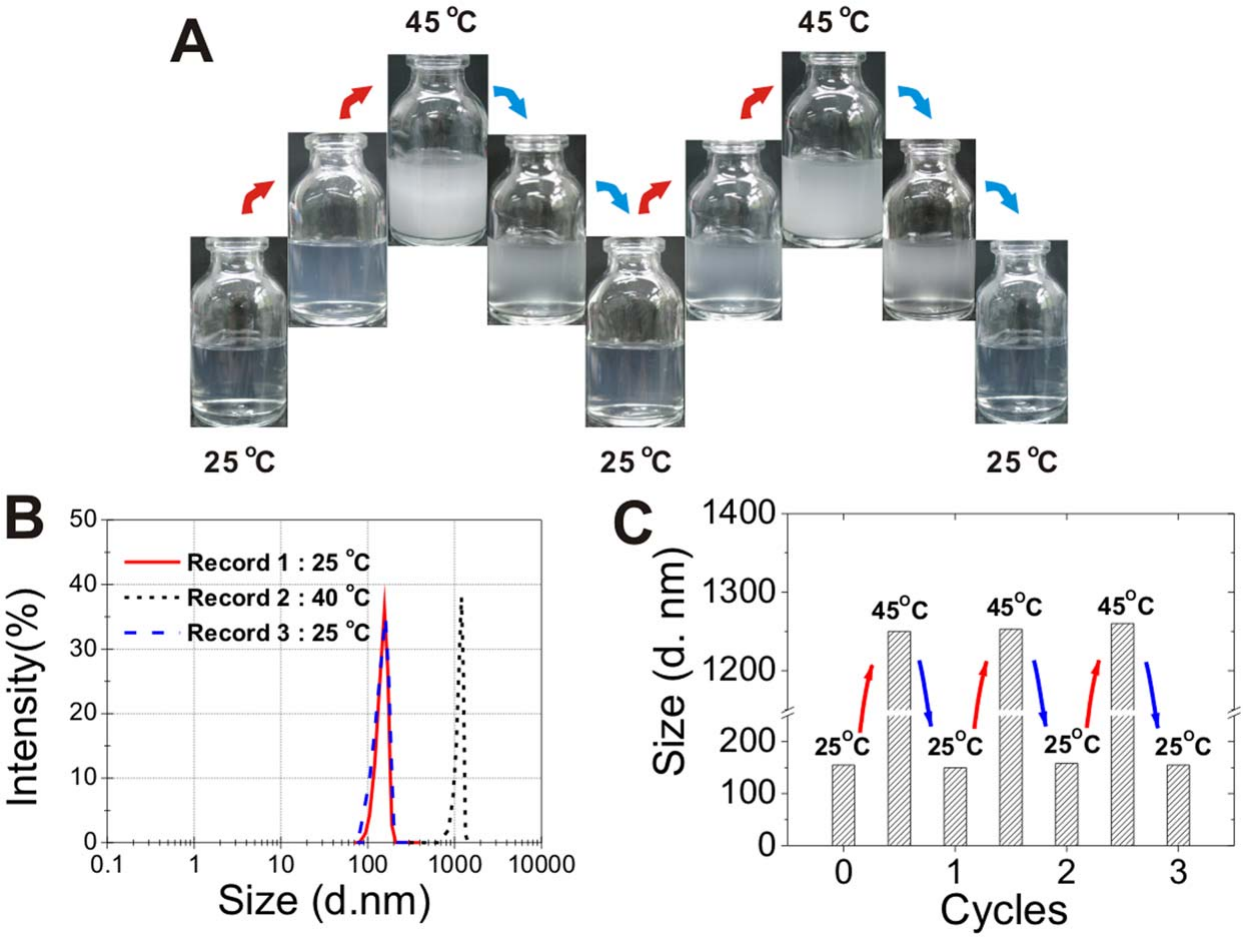
Temperature responses effect of CLGNs. (A) Pictures of CLGNs at different temperatures. (B) Size distribution of CLGNs at different temperatures measured using DLS. (C) Size changes of CLGNs at 25°C and 45°C within three cycles.

Different anion modifications could affect the temperature response of CLGNs. With the increase of temperature, the clear solutions of CLGNs turned translucent. The images of CLGNs at 25°C, 35°C and 45°C were taken and shown in Panel A of Figure 2. As the temperatures increased, the clear solutions turned translucent. While the temperatures decreased, the solutions turned clear again. These phenomena clearly showed the swelling and contracting of CLGNs. With several cycles of temperature rising and cooling, these phenomena could still be observed. This result indicated that the reversible swelling and contracting of CLGNs. The time taken for the phase transition of CLGNs was recorded as response time. Then the temperature decreased gradually to room temperature to observe if the translucent solution turned clear again. The results are listed in Table 1.

**Table 1:** Temperature-Sensitive Response for Different Anions Modified CLGNs.

Anion	Phase Transition Temperature (°C)	Response Time	Reversibility
X^−^ (2.0 mol/L)			
F^−^	40	2′26′′	√
Cl^−^	46	5′59′′	
Br^−^	47	1′28′′	
I^−^	45	6′	
NO_3_^−^	48	4′	√
CH_3_COO^−^	40	1′53′′	√
X^2−^ (1.0 mol/L)			
CO_3_^2−^	41	3′08′′	
SO_4_^2−^	45	1′46′′	
X^3−^ (0.7 mol/L)			
HPO_4_^3−^	40	2′27′′	

The concentrations of these series sodium solutions with different anions for the CLGNs surface modification were dependent on the charge number of the anions to obtain the same ionic strengths.

As the results shown in Table 1, the fluoride and acetate anion modified CLGNs clearly bear an excellent reversible temperature response. Some anions could precipitate gelatin in the CLGNs preparation step, such as CO_3_^2−^ and PO_4_^3−^. While others could be used to get CLGNs but made the CLGNs irreversible to the temperature-triggered phase transition, such as Cl^−^, Br^−^, I^−^ and SO_4_^2−^. To explain the reasons that CLGNs modified with F^−^ and CH_3_COO^−^ could exhibit excellent reversible temperature response, we hypothesized that the polarizability of the modification anions contribute to the surface properties of CLGNs. The polarizability of ions was reported increase in the following order: F^−^<CH_3_COO^−^<Cl^−^<Br^−^<I^−^ [38]. This means the F^−^ and CH_3_COO^−^ could hold electrons more tightly than other anions, while all of the counter cations for the modification were sodium. Due to the fact that anions with weak ion polarizability would have strong repulsion between each other, we hypothesized that the modification of CLGNs with F^−^ and CH_3_COO^−^ would result in a stronger repulsion at the surface of the CLGNs. When the temperature was increased, the stronger repulsion could more easily counteract the hydrogen bonds between oxygen and nitrogen atoms in CLGNs [39]. In other words, the CLGNs modified with F^−^ and CH_3_COO^−^ should have a lower phase transition temperature. Moreover, the acetate anion modified CLGNs showed a quicker response time, Thus, 1.0 mL of 2.0 mol/L sodium acetate was used as the modification solution in the preparation of reversible temperature-sensitive CLGNs.

### Morphology and Size Distribution of Cross-linked Gelatin Nanoparticles

**Figure 3:**
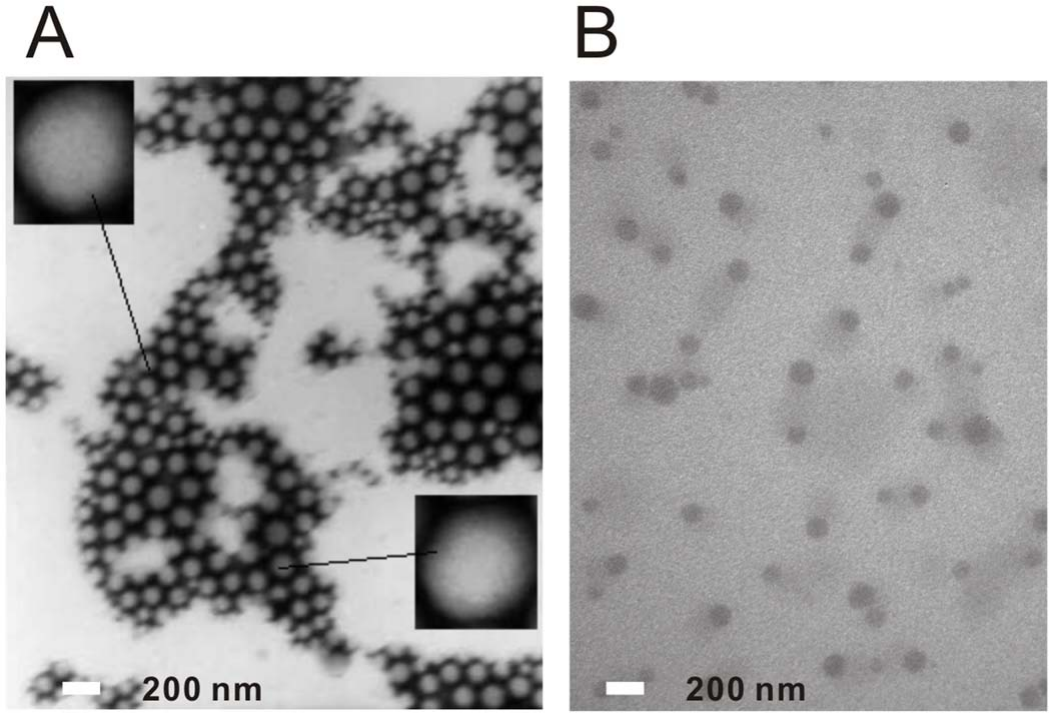
TEM image of the CLGNs. (A) negative stained by phosphotungstic acid and (B) gluocoseamylase immobilized CLGNs prepared by adsorption method.

Panel B of Figure 2 showed that the size distribution measured by dynamic light scattering at different temperatures. The results showed that at room temperature, CLGNs were monodispersed with diameter of 155±5 nm and polydispersity index (PdI) around 0.14. With the small PdI value, it is clearly known that the CLGNs were homogeneous [40], [41]. As the temperature elevated from 25 to 40°C, the diameter of CLGNs increased to 1257±24 nm while the PdI remained the same value. TEM image also showed that CLGNs were monodispersed and almost spherical in shape (SEM photo of CLGNs also confirmed this as Figure S1 shown). Most of the CLGNs had a diameter around 150 nm (Figure 3, Panel A). TEM image of glucoamylase loaded CLGNs prepared by adsorption method was also shown (Figure 3, Panel B). After the enzyme immobilization, the size of CLGNs didn’t change significantly. The PdI value measured from the TEM image was 0.18, which correspondence with the data of unloaded CLGNs. These results confirmed that during the enzyme immobilization process, the CLGNs exhibited an excellent reversible swelling property.\.

From the results of temperature-sensitive size distribution of CLGNs, it was also confirmed that the CLGNs exhibited an excellent reversible temperature-sensitive response. The increased diameter could be attributed to the swelling of the CLGNs rather than the aggregation caused by raised temperature because the constant PdI and the contracting of CLGNs as the temperature decreased to room temperature. The Zeta potential measurements of CLGNs at different temperature were also confirmed reversible swelling of the nanopaticles. With the increasing of temperature, the Zeta potential became more negative among acidic, neutral and basic conditions (Panel A in Fugre 4). These results indicated that with increased temperature, the repulsion of at the surface of the gelatin became stronger, which were according with the results listed in Table 1.

In addition, after storing under common conditions, such as in a refrigerator or at room temperature for six months, the CLGNs did not exhibit any significant change in morphology and particle size (data not shown). These indicate the stability and good fluidity of acetate modified CLGNs.

The swelling degree results of CLGNs are listed in Table 2. As the temperature elevated, the CLGNs exhibit swelling process, observed as the increased diameter at the temperature above 40°C. When the temperature decreased below 40°C, the diameter of the CLGNs got back to 155±5 nm. The results showed that the CLGNs have excellent reversible temperature-triggered swelling properties.

**Table 2:** Reversible Temperature-Triggered Swelling Degree of the CLGNs, gulocoseamylase immobilized CLGNs prepared by entrapment and adsorption methods respectively.

T (°C)	CLGNs		Enzyme Entrapped		Enzyme Adsorbed	
	Average Diameter (nm)	*SD*%	Average Diameter (nm)	*SD*%	Average Diameter (nm)	*SD*%
25	155.0	0	154.0	0	156.0	0
40	1257^a^	87.7	1261^a^	87.8	1258^a^	87.6
50	1300^a^	88.1	1298^a^	88.1	1325^a^	88.2
60	1381^a^	88.8	1315^a^	88.3	1330^a^	88.3
50	1326^a^	88.3	1296^a^	88.1	1312^a^	88.1
40	1230^a^	87.4	1250^a^	87.7	1256^a^	87.6
25	155.0	0	156.2	1.4	158.3	1.5

All the data in groups is compared with the data in 25°C.

a Data is significantly different (*P*<0.05) from the data at 25°C.

**Figure 4:**
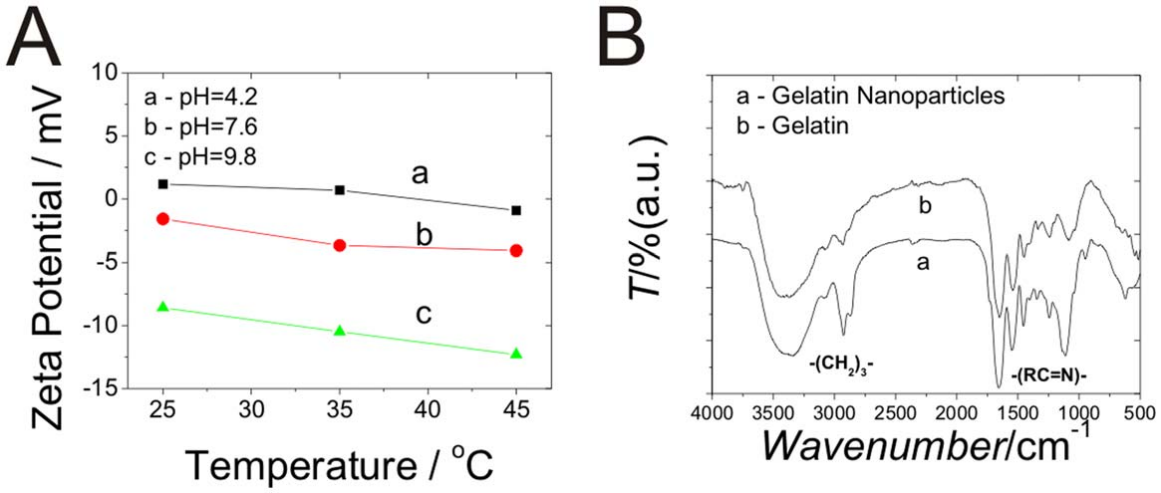
Characterization of CLGNs. (A) Zeta potential measurements values of the CLGNs under different temperature. (B) IR spectra of lyophilized CLGNs. Spectra of pure gelatin were also presented for comparison.

In Panel B of Figure 4, the IR spectra of lyophilized CLGNs are shown. Glutaraldehyde was used as a cross-linker to for a Schiff base with the amino group of gelatin. This method was widely used in biochemistry and gelatin nanoparticles preparations [42]. Comparison of the IR spectra of the CLGNs and pure gelatin shows that the strengthened IR absorption around 2900 nm^−1^ could be attributed to the stretching vibration of -(CH_2_)_3_-, which was derived from the glutaraldehyde used as cross-linking reagent. On the other hand, the strong peak of glutaraldehyde around 1750 nm^−1^ was not observed, while the intensity around 1110 and 1660 nm^−1^ increased. This confirmed the formation of Schiff base between the carbonyl group of glutaraldehyde and amino group of gelatin [43]–[45].

### Efficiency of Glucoamylase Immobilization

Glucoamylase (EC 3.2.1.3) is a biocatalyst capable of hydrolyzing α-1, 4 glycosidic linkages in raw or soluble starches and related oligosaccharides to produce β-glucose. Recent research on glucoamylase immobilization mainly focused on the entrapment of the cross-linked enzyme and covalent binding on different matrices in order to achieve industrial applications [23]. The disadvantage of these immobilization methods is the inactivation of the enzyme [16], [17]. In current study, glucoamylase was immobilized by the entrapment and adsorption methods to prevent inactivation. Furthermore, the optimum temperature range for glucoamylase is 40–70°C. This property makes it possible for the enzyme to maintain its activity during the immobilization process and perform its highest catalytic ability after its release, which was carried out above the CLGNs’ phase transition temperature (40°C).

In the first attempt to immobilize glucoamylase by the entrapment method in distilled water, the efficiency of the immobilization was 22.5% and the amount of the enzyme loaded was 2.48 mg per 100 mg CLGNs. The isoelectric point (pI) of the gelatin used in this study is 5.0, whereas the pI of the glucoamylase is around 3.0∼4.0. When the gelatin and glucoamylase were oppositely charged, the efficiency of the immobilization was predicted to increase. The experimental results showed that if gelatin was dissolved in an acetate buffer solution (0.1 mol/L, pH 4.6) instead of distilled water at the beginning step of CLGNs preparation, the efficiency of immobilization increased to 59.9% and the amount of enzyme loaded was 6.59 mg per 100 mg CLGNs.

Immobilization of glucoamylase by the adsorption method was carried out at different temperatures (25, 37 and 60°C). The efficiency of the immobilization at 25 and 37°C were 19.8% and 26.7% respectively, using the CLGNs without dialysis. When the adsorption temperature was raised to 60°C, the immobilization efficiency was as high as 82.7%, indicating that the temperature-triggered CLGNs phase transition could increase the amount of adsorbed enzyme. But in the following glucoamylase release experiment, there was no release observed for adsorbed enzyme from the CLGNs.

Another strategy using CLGNs with dialysis to adsorb glucoamylase was carried out at different temperature. The results listed in Table 3 showed that immobilization efficiency with dialyzed CLGNs were not as high as non-dialyzed ones. But in the following glucoamylase release experiments, the dialyzed CLGNs showed excellent temperature-triggered enzyme release property.

**Table 3:** Glucoamylase Immobilization Efficiency by Adsorption Method Using Dialyzed and Non-dialyzed CLGNs at Different Temperature.

Temperature (°C)	Non-dialyzed CLGNs *η_L_*(%)	Dialyzed CLGNs *η_L_*(%)
25	19.8	8.3
37	26.7	6.7
60	82.7 a, b	24.7 a, b

a Data is significantly different (*P*<0.05) from the data of at 25°C;

b Data is significantly different (*P*<0.05) from the data at 37°C.

The reasonable explanation for the immobilization efficiencies of dialyzed and non-dialyzed CLGNs also rely on that the glutaraldehyde used as cross-linking reagent was remained in the solution of non-dialyzed CLGNs. In the immobilization step, glutaraldehyde could react with the glucoamylase forming covalent linkage between gelatin and glucoamylase, which resulted in the higher immobilization efficiency. But the covalently linked enzyme was hardly to be released or the covalent linkages deactivated the glucoamylase. On the other hand, when perform the immobilization using dialyzed CLGNs, the glucoamylase was physically adsorbed at the gelatin matrix. Although the immobilization efficiency was only around 25%, the temperature-triggered enzyme release property of the dialyzed CLGNs was satisfactory.

Various amounts of dialyzed and lyophilized CLGNs powder (20, 30 and 60 mg) were used to investigate the optimum conditions for immobilization. The immobilization efficiency was determined as 17.8%, 24.7% and 24%, respectively. The results showed that the increased amount of the CLGNs powder improved the immobilization efficiency. However, when the added gelatin powder exceeded 30 mg, the efficiency did not increase any further. Thus the optimum amount of CLGNs powder for enzyme adsorption was determined to be 30 mg.

The concentration of glucoamylase also had an effect on the immobilization efficiency. 3 mL of solutions with different glucoamylase concentrations were used to carry out the adsorption studies. The concentrations of these solutions varied from 0.5, 1.0 and 2.0 mg/mL with the resulting immobilization efficiencies as 23.8%, 24.7% and 12.2%, respectively. The decreased immobilization efficiency with 2.0 mg/mL glucoamylase solution indicated that the adsorption of enzyme saturated at the higher concentration. This means at low level of enzyme concentration, the more enzyme added, the more it could be adsorbed. When the enzyme concentration was high enough, the CLGNs adsorbed enzyme in its capacity completely. So the concentration of glucoamylase was set at 1.0 mg/mL in the subsequent experiments for the temperature-triggered enzyme release.

### Temperature-Triggered Glucoamylase Release

Enzyme immobilization by entrapment showed no release of enzyme even when the temperature was raised to 60°C. So this immobilization method could not be used for temperature-triggered enzyme release. The reason for the high immobilization efficiency and no release in the entrapment method for enzyme immobilization could be attributed to the glutaraldehyde added in the CLGNs preparation. Glutaraldehyde not only cross-links the amino acid residues on the gelatin chain, but also with those on the glucoamylase. The cross-linking between gelatin and glucoamylase resulted in the high immobilization efficiency. On the other hand, the firm cross-linking could also inhibit the release of glucoamylase.

**Figure 5:**
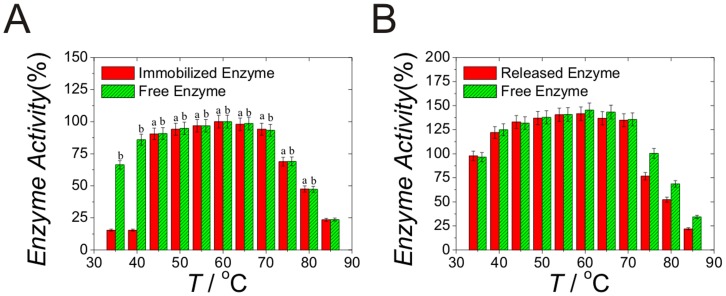
Activities of the glucoamylase at different temperature. (A) 30 mg of dialyzed and lyophilized powder was added into 3 mL of 1.0 mg/mL glucoamylase solution to perform the adsorption of enzyme at 60°C for four hours and then at 25°C for one hour. After centrifugation and washing process to remove free enzyme, the sedimentation of CLGNs were incubated at different temperature to determine the activities of released enzyme. The activities of free glucoamylase were also calculated at the same condition for comparison. (B) Comparison of enzyme activities for the released glucoamylase at 60°C and free enzyme at temperature range of 30–85°C a: Data are compared with the data of released enzyme at 40°C and significantly different (*P*<0.05) from that data; b: Data are compared with the data of free enzyme at 85°C and significantly different (*P*<0.05) from that data.

Temperature-triggered enzyme release for the immobilized glucoamylase by adsorption method with dialyzed CLGNs was also examined. The results are shown in Panel A of Figure 5. For comparison, the activities of the free enzyme at the same temperature are also shown. At temperatures below 40°C, the activities of glucoamylase in the CLGNs solution were very low, indicating that the immobilization was effective and there was no enzyme released. When the temperature was set above 40°C, higher than the phase transition temperature of the CLGNs, the immobilized enzyme was immediately released and showed recovered activities. The release efficiency examined at 60°C was 99.3%, indicating that the temperature-triggered enzyme release was complete.

In order to confirm the fact that the immobilized enzyme could not perform its catalytic ability, the experiment of mixing CLGNs and glucose were carried out using blank CLGNs and enzyme immobilized ones at the temperature blow 40°C. The results showed that neither the blank CLGNs nor the glucoamylase immobilized ones show evident enzyme activities. The conclusion is the immobilized glucoamylse could not catalyze the hydrolysis of glucose. In other words, the immobilization by CLGNs prevented the catalytic abilities.

Based on the experiments of temperature-triggered enzyme release using dialyzed CLGNs by adsorption method, we hypothesized that compact CLGNs could be formed when the temperature is below 40°C because of the hydrogen bonds between oxygen and nitrogen atoms in the amino acid residues. When the temperature was raised above 40°C, the hydrogen bonds broke and the CLGNs swelled, which was observed as the phase transition. The size of the CLGNs dramatically increased, and the mesh size at the surface of the nanoparticles also enlarged. At this phase, the glucoamylase could easily enter and come out from the CLGNs. When the temperature was decreased below 40°C, the hydrogen bonds were formed again and the CLGNs quickly shrank back to their original size. This process completes the adsorption of glucoamylase. After immobilization was completed, the centrifugation and washing step removed the free enzymes remaining in the solution. When the temperature was elevated again, the swelling of the CLGNs made it possible for the complete release of immobilized glucoamylase.

By comparison of the activities for the released glucoamylase at 60°C and free enzyme at temperature range of 30–85°C, there were no significant differences (Panel B in Figure 5). These results indicated that the immobilization and release process had no major effect on the activities of glucoamylase. The different enzyme activities for the released and free glucoamylase observed below 40°C in Panel A of Figure 5 evidently derived from the immobilization of glucoamylase in the CLGNs which could not catalyzed the hydrolyzation of starch while the free glucoamylase could.

Attempts to achieve reversible immobilization of glucoamylase after the temperature-triggered release were also carried out. After the release of glucoamylase above the phase transition temperature, the temperature was reduced to 25°C for four hours for reversible immobilization. The solution was centrifuged for 10 min under 18000 rpm for the sedimentation of CLGNs, as described previously. The sediment was then re-dissolved in an acetated buffer (0.1 mol/L, pH 4.6) and the temperature was raised to 60°C for enzyme release determination. Unfortunately, reversible immobilization and release were not observed. This may be because the immobilization efficiency by adsorption was not high enough (24.7%) for the re-adsorption. Future researches will focus on improvement of immobilization efficiency to explore the reversible immobilization and release for enzymes.

### Conclusion

The presented study introduced a novel strategy for the preparation of acetate modified CLGNs which showed a reversible temperature-triggered swelling. Morphology results showed that the CLGNs were regularly spherical, with average diameters of about 155±5 nm. As well, the CLGNs exhibited favorable dispersivity. When the ambient temperature was above 40°C, the diameters of CLGNs could increase to over 1 µm in a few minutes, and their reversible temperature-sensitive properties are fine. By the adsorption method, glucoamylase could be easily immobilized in the CLGNs with loading efficiency of 24.7% and 3.14 mg glucoamylase per 100 mg CLGNs, respectively. When the system temperature was higher than 40°C, which was the phase transition temperature of acetate modified CLGNs, the absorbed glucoamylase was released completely. The activity of released glucoamylase was not affected by the immobilizationn and it could function as a free enzyme during the catalytic reaction. This system may be an effective alternative for enzyme immobilization used in biotechnology.

## Supporting Information

Figure S1 **SEM image of the gluocoseamylase immobilized CLGNs prepared by adsorption method.**
(TIF)
