# Supplementary figures and images for: Temperature-Triggered Enzyme Immobilization and Release Based on Cross-Linked Gelatin Nanoparticles
Source: PLoS One. 2012 Oct 10;7(10):e47154. doi: 10.1371/journal.pone.0047154 (PMC3468439; doi:10.1371/journal.pone.0047154)

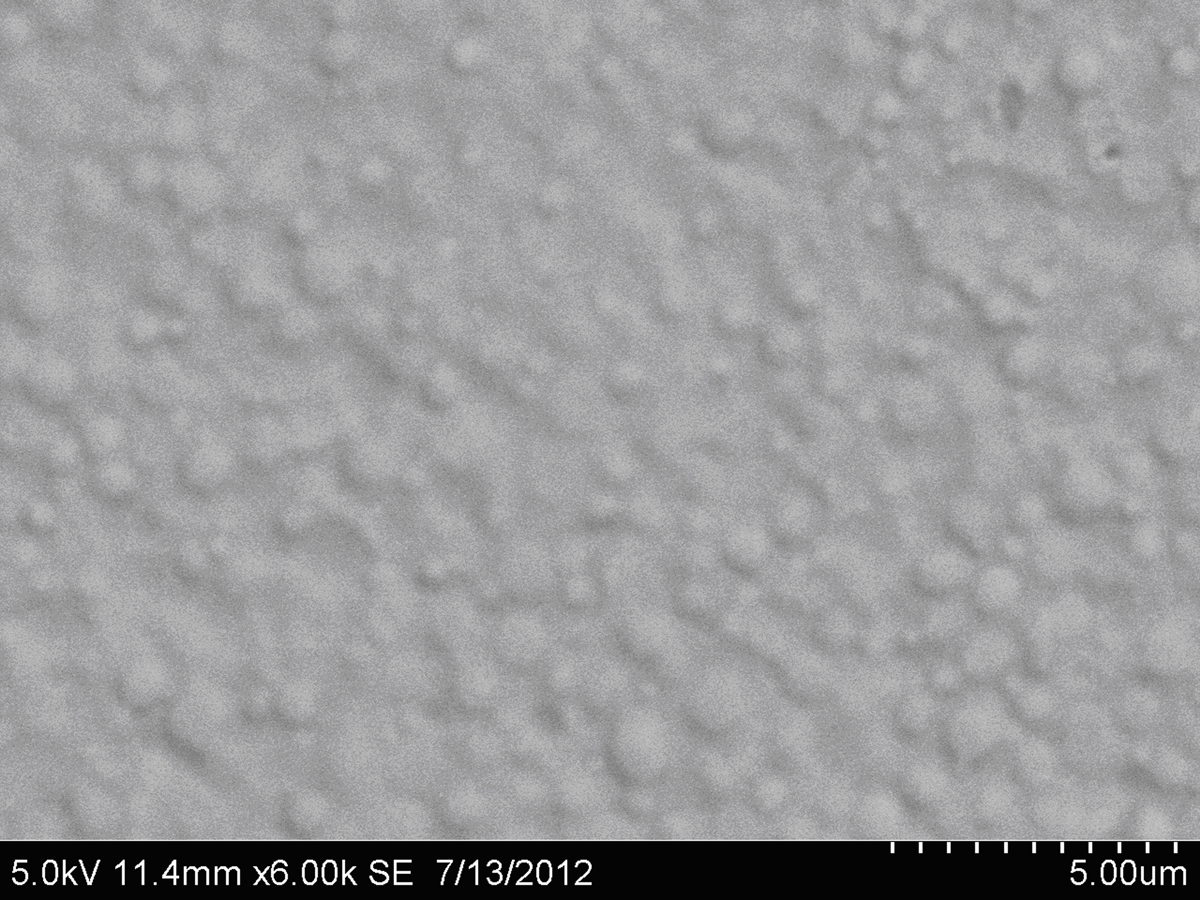

Supplement: Figure S1 — SEM image of the gluocoseamylase immobilized CLGNs prepared by adsorption method. (TIF) [file pone.0047154.s001.tif]
